# Inversion of interlayer scale using dynamic data of production wells in bottom water reservoirs: Sangtamu Oilfield, Tarim basin

**DOI:** 10.1038/s41598-025-86149-x

**Published:** 2025-01-24

**Authors:** Mengjiao Dou, Shaohua Li, Wanjiang Bian, Huan Wang, Lunjie Chang, Zhengjun Zhu, Jun Li

**Affiliations:** 1 School of Geosciences, Yangtze University, Wuhan, 430100 China; 2 Exploration and Development Research Institute, PetroChina Tarim Oilfield Company, Korla, 841000 China

**Keywords:** Interlayer scale, Bottom water reservoir, Dynamic information, Bottom water coning, Geologic knowledge database, Geology, Geophysics, Sedimentology, Crude oil

## Abstract

The target reservoir is a typical blocky bottom water reservoir. There are several interlayers of varying scales inside it, which impact the characteristics of the oil production and water breakthrough curves of the wells in the water flooding development oilfield, resulting in strong heterogeneity within the reservoir. The Sangtamu Oilfield has an average well spacing of approximately 600 m, causing a challenge in accurately identifying the range of small-scale interlayer spreading. This, in addition, challenges the subsequent 3D geological modeling process. This paper proposes a method to accurately characterize the size of various interlayers in a reservoir by using production dynamics data from bottom water reservoirs to invert the interlayer scale. By classifying the water breakthrough curve styles derived from theoretical testing, the range of reservoir internal interlayer scales is compared and inverted based on the actual water breakthrough styles of wells in the Sangtamu oil field. In the process, many interlayer scales derived from various types of geologic data acquisition were combined to form a quantitative geologic knowledge database of interlayers that synthesizes both dynamic and static data. This method has been applied in the Tarim Basin’s Sangtamu Oilfield, where the reservoir numerical simulation history was used to fit the model. This resulted in an overall fitting coincidence rate of 96% in the Sangtamu Oilfield and a single-well fitting coincidence rate of over 90% in well LN14. This method offers a new viewpoint on characterizing the interlayer’s scale in the area of the non-dense well network.

## Introduction

Although blocky bottom-water reservoirs are abundant reserves in China, they are challenging to sustain due to their strong heterogeneity and rapid rise in water content^1–3^. Bottom water coning is currently the primary challenge to developing blocky bottom water reservoirs^4,5^. However, the interlayer has an obvious inhibiting effect on suppressing bottom water coning, which can improve the recovery rate^6,7^. The extraction of remaining oil within the reservoirs becomes vital in the middle and late stages of oil and gas field development. Consequently, the characterization and scale representation of the reservoir’s internal interlayer have received increasing attention in recent years^8,9^. Numerous studies have revealed that the reservoir contains large number of interlayers. The interlayer’s thickness and planar scale vary greatly, significantly impacting the water-injected oilfield’s oil-water flow and seepage path. To some degree, it also decides where the primary region of residual oil distribution is located^10–12^. Hence, it is of great significance to carry out the study on the characteristics and scale of the interlayer to analyze the distribution of remaining oil and to take effective measures for digging up the potential.

Reservoir engineers have been dedicated to analyzing the primary elements influencing bottom water coning in reservoirs and accurately forecasting the timing of water breakthroughs in wells in bottom water reservoirs^2,13,14^. These have emerged as crucial issues in recent years. Additionally, several academics have fitted the prediction formula for the time of water breakthrough in bottom-water reservoirs^13,15–19^, and some of these formulas have been used to demonstrate the interlayer’s actual spreading range^20^. Reservoir numerical simulation is primarily employed to evaluate the diffusion law of mass transfer between fracture and matrix^21^, simulate CO miscibility in shale reservoirs^18^, analyze oil and gas production rates and reservoir mechanical response^19^, analyze the impact of hydrate reservoir properties on gas production^22^, simulate geothermal heat ^27^, and orthogonally simulate the distribution characteristics of different architectural units in a 3D geological model. Traditional geological study employs data from individual wells to identify the thickness of the interlayer^24^, quantitatively establish empirical formulas, and assess the interlayer’s scale, thickness, frequency of development, and distribution pattern with continuous well profiles and architectural patterns^23^. The range of interlayer spreading can be determined by utilizing various methods such as variance function calculations ^27,30^, resistivity logging calculations^26^, 3D seismic data identification ^30,33^, modern sedimentation, and field outcrop measurements^29,30^. Empirical formulas for width and thickness can be used to fit the interlayer scale, and a geologic knowledge database can be established to guide the subsequent 3D geologic modeling process. However, most static data is presently used for estimating the interlayer parameters’ scale, and there are few applications of production dynamic data for reservoir numerical simulation to invert and obtain interlayer scale. We consider combining dynamic and static data to obtain the scale of the interlayer comprehensively and establish a quantitative geological knowledge database to guide the geological modeling.

This paper proposes a method to reverse the scale of the interlayer by using dynamic information about producing wells in response to the issues mentioned above. Initially, an analysis is conducted on the primary factors that influence the water cone velocity in the interlayer. A total of 72 test groups were performed using an orthogonal experimental design. The test results are then compared and classified to analyze the distinct characteristics of various interlayer water breakthrough curves. The planar scale of the interlayer is predicted based on the inversion of the water breakthrough curve pattern by comparing the test results with the actual dynamic oil and water production patterns of various oil wells in the study area. The interlayer scale obtained from the geostatic data is combined to establish the quantitative geologic knowledge database. The interlayer parameters were obtained from the geological knowledge database. The interlayer was modeled using an object-based method with application in the Tarim Basin’s Sangtamu oil field. According to the history matching of reservoir numerical modeling, the total reservoir coincidence rate is better than 90%. The range of interlayer scales obtained with this method is consistent with geological knowledge and the established interlayer model, which more accurately predicts the interlayer between wells and characterizes the scale of the over-well interlayer. This provides a scientific basis for the distribution characteristics of the remaining oil in bottomwater reservoirs.

## Geological setting

**Fig. 1:**
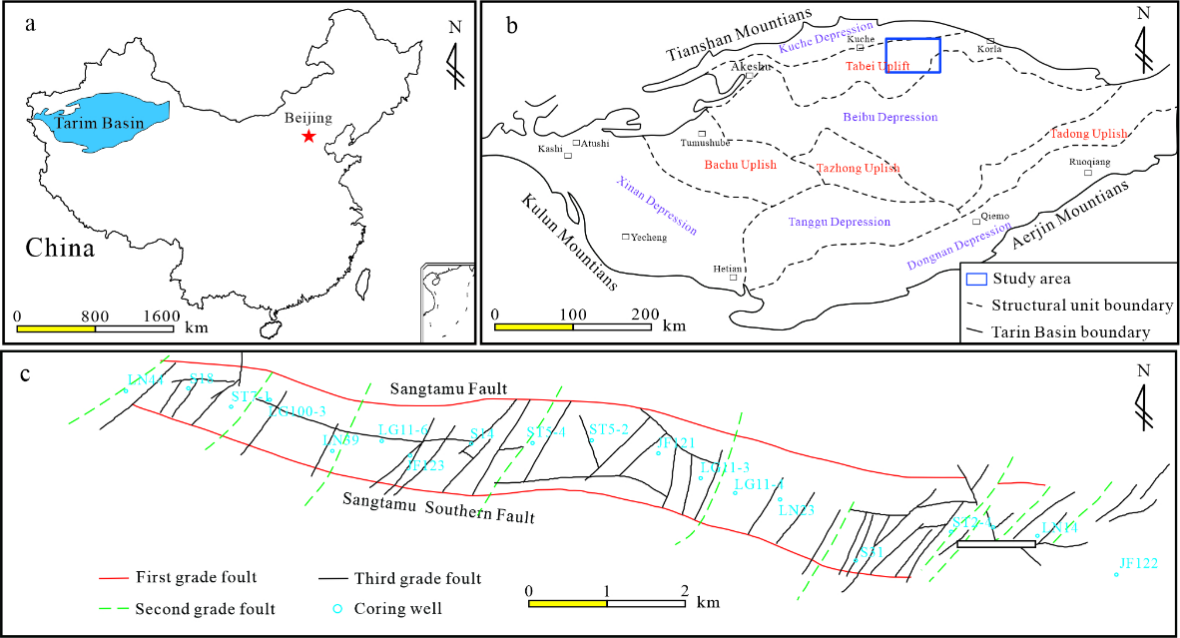
Geographic location distribution of the Sangtamu oilfield (after Dou et al.^31^, 2023). (a) Geographical location of Tarim Basin; (b) Structural map of Tarim Basin; (c) Wells location of Sangtamu oilfield.

The Tarim Basin located in the southern part of the Xinjiang Uygur Autonomous Region (Fig. 1a). The structural location of the Sangtamu oil field is on the Sangtamu buried hill drape anticline belt of the Lunnan Slope in the south of Tabei Uplift’s (Fig. 1b)^31^. It is a long and narrow anticline belt with a length of approximately 24 km, spreading in an east-west direction. The anticline is split into seven oil-bearing fault blocks by a sequence of minor north-south-oriented faults, forming traps that gradually drop to the west^32^ (Fig. 1c). Its reservoir type is a large bottom water reservoir with sufficient bottom water energy and a water body multiplier of 267.52. Each block has a different oil-water interface with an upward elevation from east to west.

**Fig. 2:**
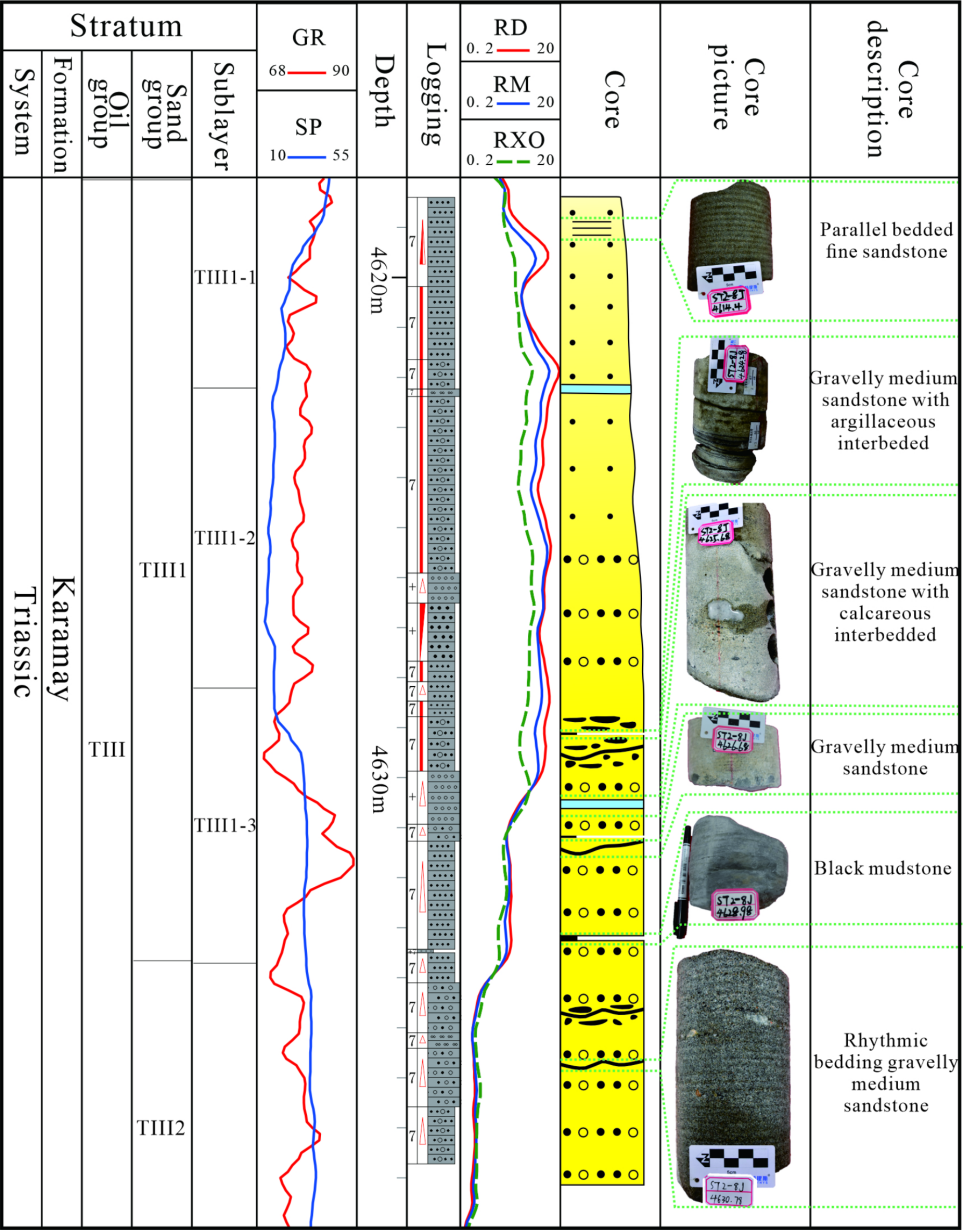
Comprehensive stratigraphic column of the Sangtamu oilfield.

The Sangtamu oil field Triassic can be subdivided into three oil groups: TI, TII, and TIII. The sedimentation period of the TIII oil group was primarily characterized by the deposition of braided river deltas, which were influenced by an abundant water supply and a humid climate. The TIII oil group is formed by superpositioning thick sandstone bodies and channels of several phases^31^. It is thought to be formed as “sand-encased mud,” with calcareous, muddy, and physical interlayers developing inside the strongly heterogeneous reservoir. The TIII oil group is divided into three sand groups: TIII1, TIII2, and TIII3. The TIII1 Sand Formation is the primary target formation for this study (Fig. 2). The target sand formation has good physical properties, with an average porosity of 20.6% and an average permeability of 677.6 × 10^−3^ μm^2^, which belongs to the medium-porosity and medium-high permeability reservoirs. The sand body inside the reservoir is well-developed, and the distribution of internal interlayers is irregular and exhibits significant variation between different blocks. The oil-water contact of the LN14 block in the study area is 3694 m, and the distribution range of the perforation section is 4609–4621 m.

## Data and methods

### Data sources

The TIII1 sand formation of the Sangtamu oil field, one of the richest oil-bearing regions in the Tarim Basin, is the study’s target. The work area spans approximately 23 km in an east-west direction and 5.3 km in a north-south direction. Various geological data, including core, logging curve, architecture interpretation, and production dynamics, have been collected from 72 drilling wells.

### Grid data

The production well was positioned in the center of the geological model, the top of the formation was perforated, and the well was produced at a constant liquid volume according to the testing requirements. The reservoir is partitioned into 100 × 100 × 80 grids, with layers 61–80 representing water layers and grids 1–60 representing oil layers. The reservoir’s top, bottom, and all surrounding areas have closed boundaries. Layers 27–29 provide a distribution of perforation positions. There are 8,000,000 grids altogether, and the grid step lengths in the X, Y, and Z directions are 10 m, 10 m, and 0.4 m, respectively.

### Reservoir parameters

Table 1 exhibits the parameters of the layer and fluid. Table 2 provides the PVT data, and the oil-water relative permeability data are shown in Table 3.

**Table 1:** Parameters of oil layers and fluids.

Parameters	Data	Parameters	Data
Oil layer depth/m	4000	Underground crude oil viscosity/(mPa·s)	1.39
Oil layer thickness/m	30	Formation crude oil density/(g·cm^−3^)	0.7457
Bottom water thickness/m	240	Formation water viscosity/(mPa·s)	0.36
Crude oil density(20.0 °C)/(g·cm^3^)	0.834	Formation water density/(g·cm^−3^)	1
Bubble point pressure/Mpa	17.57	Oil volume factor	1.2006
Reservoir pressure/Mpa	43.2	Original oil-water interface/m	4030
Reservoir temperature/°C	103.06	Porosity/%	0.22
Initial water saturation/%	20	Permeability/10^−3^ μm^2^	40-200

**Table 2:** PVT data.

Dissolved gasoline ratio (m^3^/m^3^)	Saturation pressure of crude oil (Mpa)	Oil volume factor	Crude oil viscosity (mPa·s)
0	0	1.0869	1.76
14	3	1.1234	1.45
24	6	1.1526	1.27
36	9	1.1788	1.15
47	12	1.2036	1.1
58	15	1.2274	1.07
69	17.57	1.2489	1.06
/	49.65	1.1941	1.39

### Methodology

**Fig. 3:**
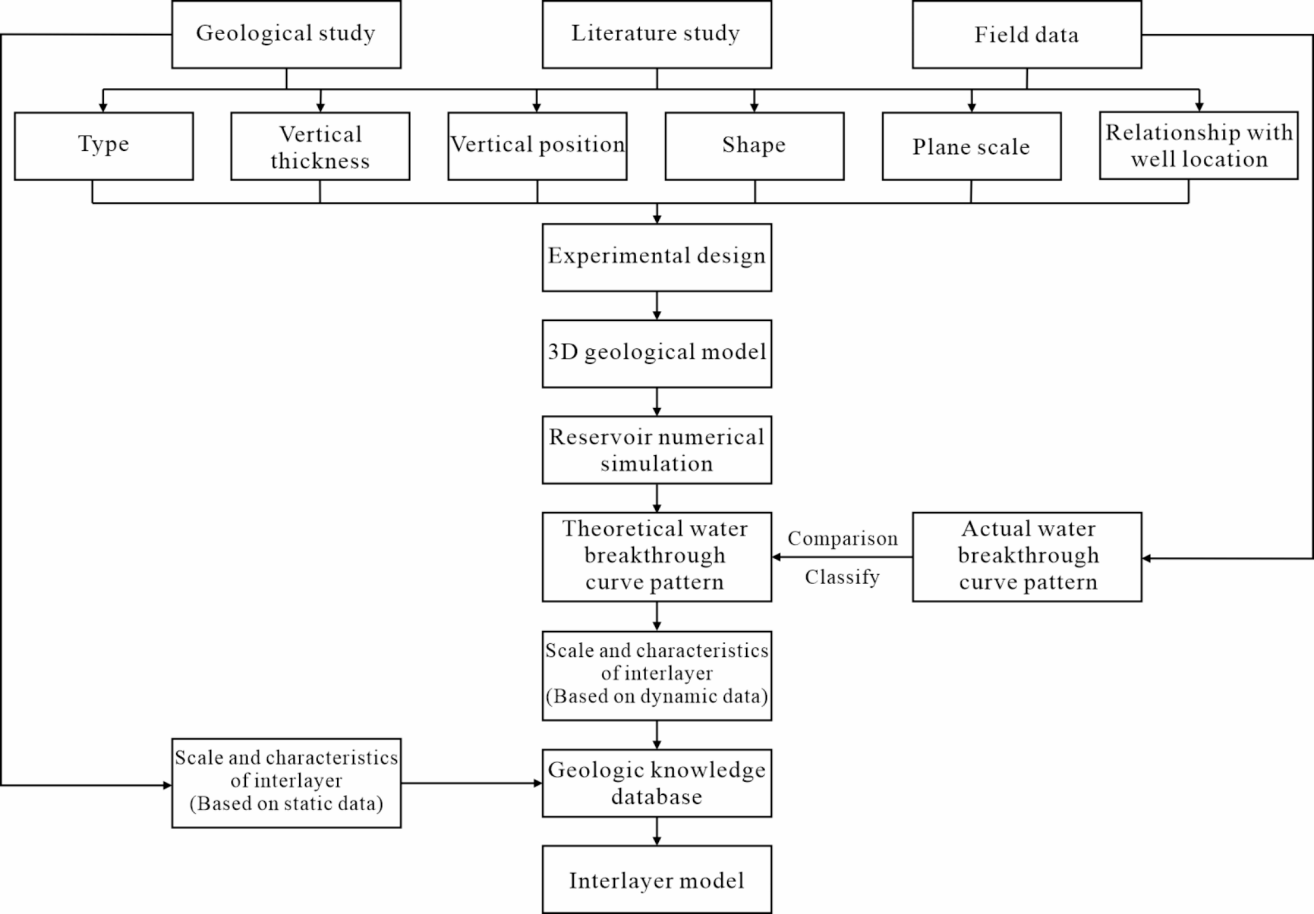
Process diagram for inverting interlayer scale using dynamic data of production wells in bottom water reservoirs.

This paper presents a method for inverting the interlayer’s scale based on dynamic data from producing wells (Fig. 3). First, we analyzed the primary factors influencing water cone velocity that are affected by the interlayer of the bottom water reservoir. We set up 72 multilevel and multifactorial tests using an orthogonal experimental design. Second, to forecast the interlayer’s planar characteristics and scale range, we compared and inverted the theoretical test water production curves with the characteristics of the actual oil and water production curves from multiple wells in the study area. Finally, based on the inversion of the water breakthrough curve and various geological data, a comprehensive quantitative geological knowledge database for interlayers is established, which combines dynamic and static data. An object-based method was applied to establish an interlayer model for the TIII1 sand formation in the Sangtamu oil field of the Tarim Basin, with the interlayer scale obtained from the geologic knowledge database. The accuracy of the interlayer scale was validated through reservoir numerical simulation and history simulation.

**Table 3:** Oil-water relative permeability data.

Water saturation (S_w)_	Water-phase relative permeability (K_rw)_	Relative permeability of oil phase in the coexistence of oil and water (K_row_)	Oil water capillary pressure (*P*_cwo_)
0.45	0	1	0.05
0.477	0.002383	0.776573	1*
0.504	0.010216	0.58535	1*
0.531	0.023937	0.42485	1*
0.558	0.043797	0.29347	1*
0.585	0.069977	0.189465	1*
0.612	0.102622	0.110903	1*
0.639	0.141849	0.055602	1*
0.666	0.187763	0.021012	1*
0.693	0.240453	0.003981	1*
0.72	0.3	0	1*
1	1	0	0

## Water curves morphological characteristics of different types of interlayers

### Analysis of influencing factors

In bottom-water reservoirs, different sources of uncertainty variables influence the bottom-water cone velocity ^33^. Six variables of the interlayer affecting the bottom water cone velocity were chosen in the tests related to the large bottom water reservoir in the Sangtamu oil field, taking account of the actual conditions of the work area and the design of the experimental design table: the type of interlayer, the location, the morphology, the vertical thickness, the planar scale, and the relationship with the well location. Each variable will be discussed individually below.

#### Interlayer types

**Fig. 4:**
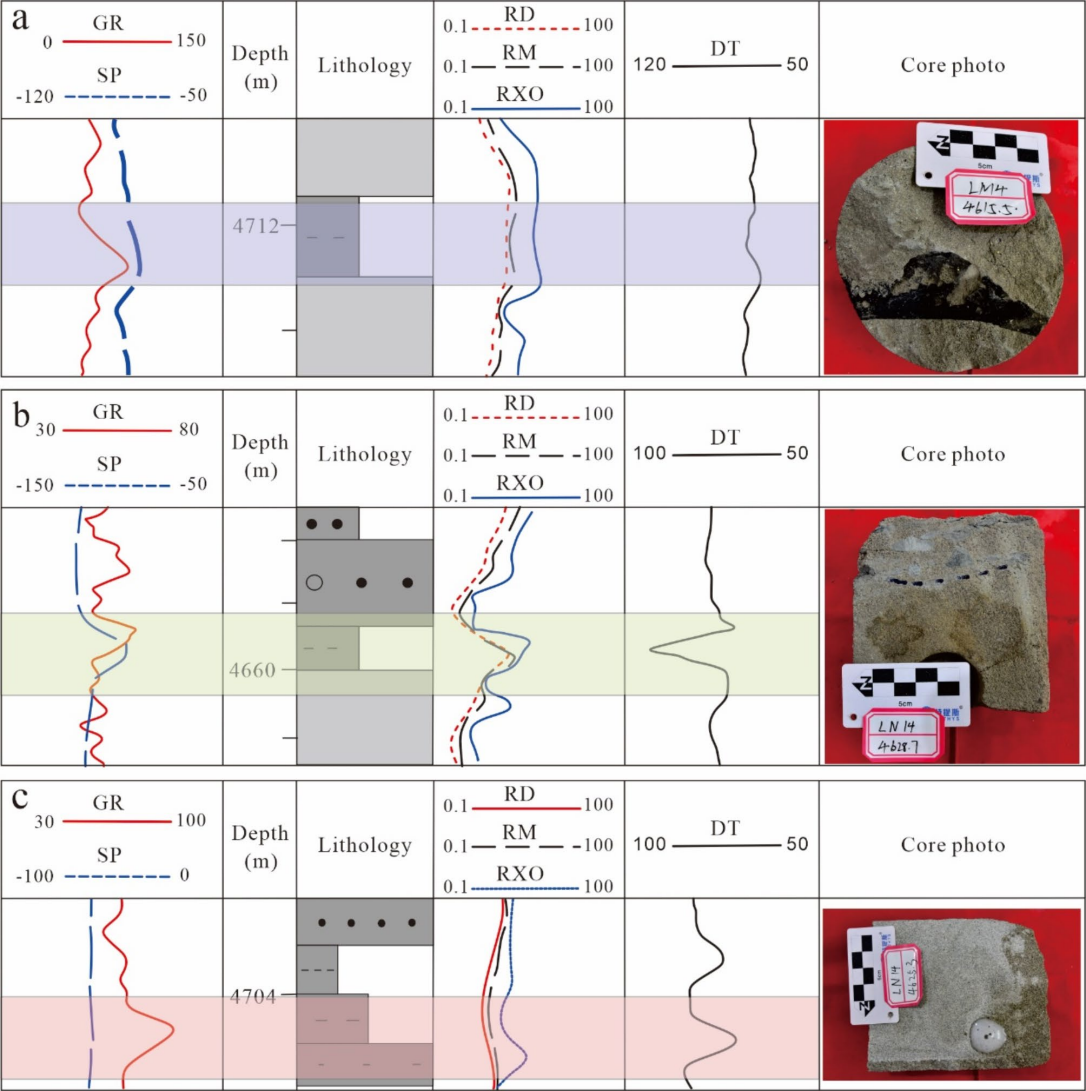
Different types of interlayers. (a) Physical interlayer; (b) muddy interlayer; (c) calcium interlayer.

Three types of interlayers have been classified in the study area: muddy, calcareous, and physical (Fig. 4). These were identified by combining the facies diagrams of individual wells and summing the response characteristics of logging curves. Each type of interlayer generally has a thickness of 0.5–2.5 m, according to the development of the interlayer in each well based on the classification results. Relying on a combination of three techniques—the oil-bearing occurrence method, the empirical statistical method, and the capillary pressure method—the lower standard of physical properties of the Triassic reservoirs in the Sangtamu area reveals that the lower limit of porosity is 12%, and the lower limit of permeability is 3 mD. In the previous study, the interval of porosity and permeability values of different types of interlayer cores was obtained based on the results of whole-rock quantitative detection by X-ray diffraction in the TIII1 oil group of the Sangtamu oil field. The interval of porosity and permeability values of well logs in the TIII1 oil group in Sangtamu oil field was obtained based on the results of secondary interpretation of the log curves. These two types of data were provided by Tarim Oilfield Exploration and Development Research Institute of PetroChina. The results of the core and logging curve measurements were synthesized to determine the range of the physical properties of different types of interlayers in this study. It is worth mentioning that there is some overlap in the physical property intervals of the three different types of interlayers, both in laboratory measurements and log curve interpretation model calculations. This study aims to amplify the impact of different types of interlayers on production conditions in an orthogonal experimental design, thus using a fixed and possible value to distinguish the distribution range of physical properties of different types of interlayers.

(i) Physical interlayer.

Physical interlayers in the reservoir have higher mud content, finer lithology, poorer physical qualities, and lower permeability characteristics, all of which have a barrier effect on gas and oil transportation. Physical interlayers reflect an abnormal return of the logging curve’s spontaneous potential curve (SP), generally between 1/4 and 3/4, with an increase in the value of the gamma-ray curve (GR) (Fig. 4a). Physical interlayers are mainly developed in the transitional zone of the facies transition, such as retention sediment at the bottom of the channel and the side edge of the channel. These are primarily deposited by fine-grained sediments after the weakening of water flow energy. This study determined that the physical interlayer porosity had a minimum of 8.5%, a maximum of 12%, and an average of 10.5%. Similarly, the permeability had a minimum of 0.7 mD, a maximum of 3 mD, and an average of 1.7 mD.

(ii) Muddy interlayer.

Fine-grained silty mudstones and muddy siltstones are the primary parts of muddy interlayer lithologies, which means these lithologies have very low porosity and permeability and are typically impermeable. Logging curves exhibiting low resistivity, high acoustic differential time (DT), abnormal spontaneous potential curve return, generally greater than 1/2, and an associated increase in the gamma-ray value are characteristic of muddy interlayers (Fig. 4b). The muddy interlayer is mainly formed at the interface between the two sets of sand bodies. It is associated with the abandonment and migratory deposition of the subaqueous distributary channel. In this study, the muddy interlayer’s minimum porosity was set at 5%, its maximum porosity at 8.5%, and its average at 6.5%. Similarly, the permeability minimum was set at 0.19 mD, its maximum at 0.7 mD, and its average at 0.5 mD.

(iii) Calcareous interlayer.

Calcareous interlayer lithologies are generally calcareous sandstones and calcium-bearing sandstones with fine grain size, resulting in extremely low porosity and permeability for this type of interlayer. A small acoustic differential time (DT) and increased density values reflect the calcareous interlayer in the logging curve. The calcareous sandstone has abnormally high resistivity values, and both porosity and permeability are below the absolute lowest limit of reservoir physical properties, with isolation near to complete isolation. Calcium-bearing sandstones exhibit high flushed zone resistivity (RXO) values, which provide a more substantial barrier compared to physical interlayers but do not achieve a complete barrier (Fig. 4c). The genesis of the calcareous interlayers, which are irregularly distributed and randomly distributed within the sand body, is associated with the non-uniformity of diagenesis, involving carbonate cementation and dissolution. The calcareous interlayer porosity in this study had a minimum of 0 and a maximum of 5%, with an average of 2.5%. Permeability had a minimum value of 0.001 mD, a maximum value of 0.19 mD, and an average value of 0.1 mD.

#### Vertical thickness of interlayer

**Fig. 5:**
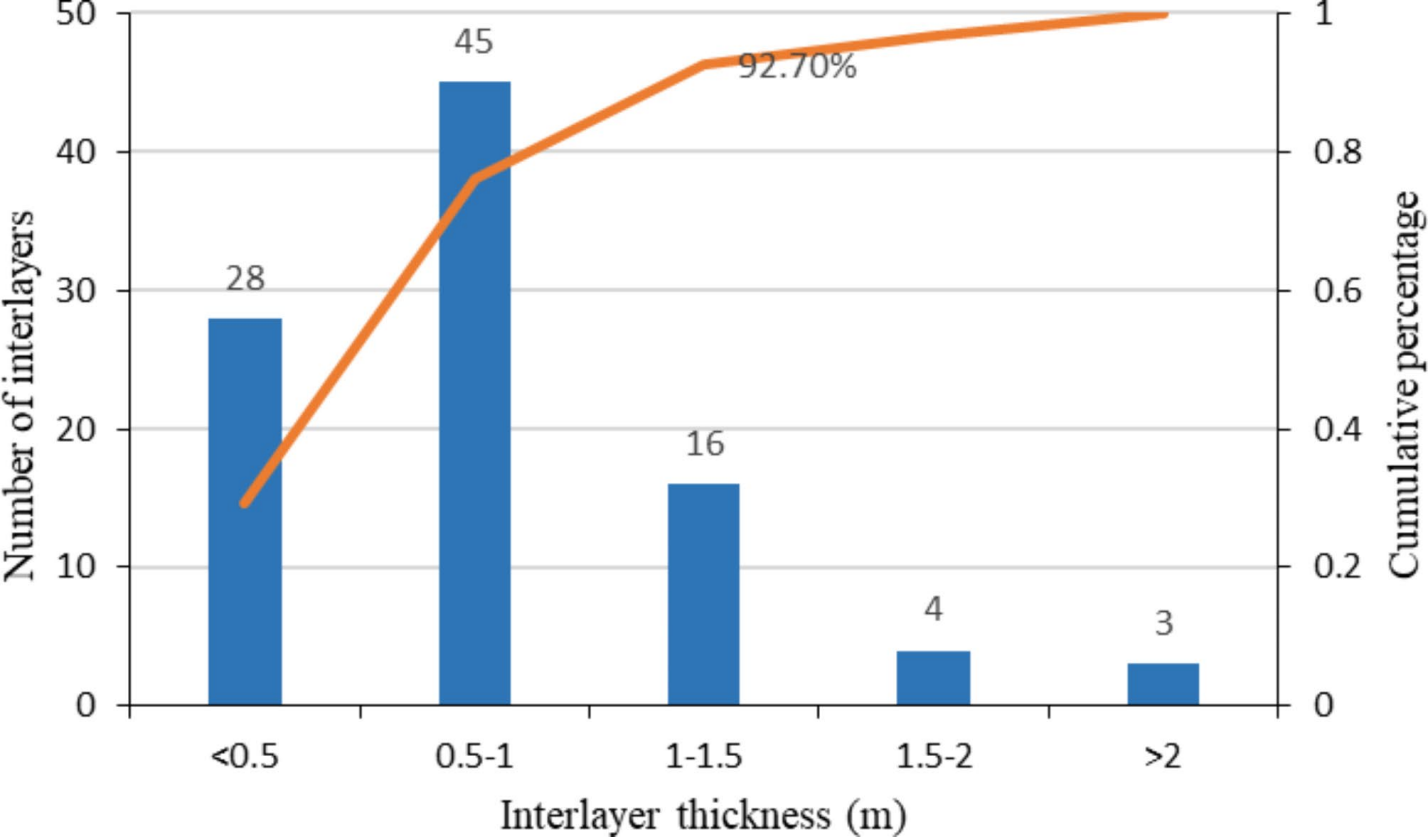
Statistics on the number and proportion of interlayers in different thickness intervals.

The degree of return of the logging curve may be used to estimate the interlayer thickness^34^. The statistics on the development of interlayers in each well in the entire area revealed that 96 interlayers were developed in the target formation in the study area (Table 4), with 29.17% having a thickness of less than 0.5 m, 46.88% having a thickness of between 0.5 and 1 m, and 92.7% having a thickness of less than 1.5 m (Fig. 5). We classified the interlayer into two types, thin and thick, depending on thickness since the model’s longitudinal grid step length is 0.4 m.

**Fig. 6:**
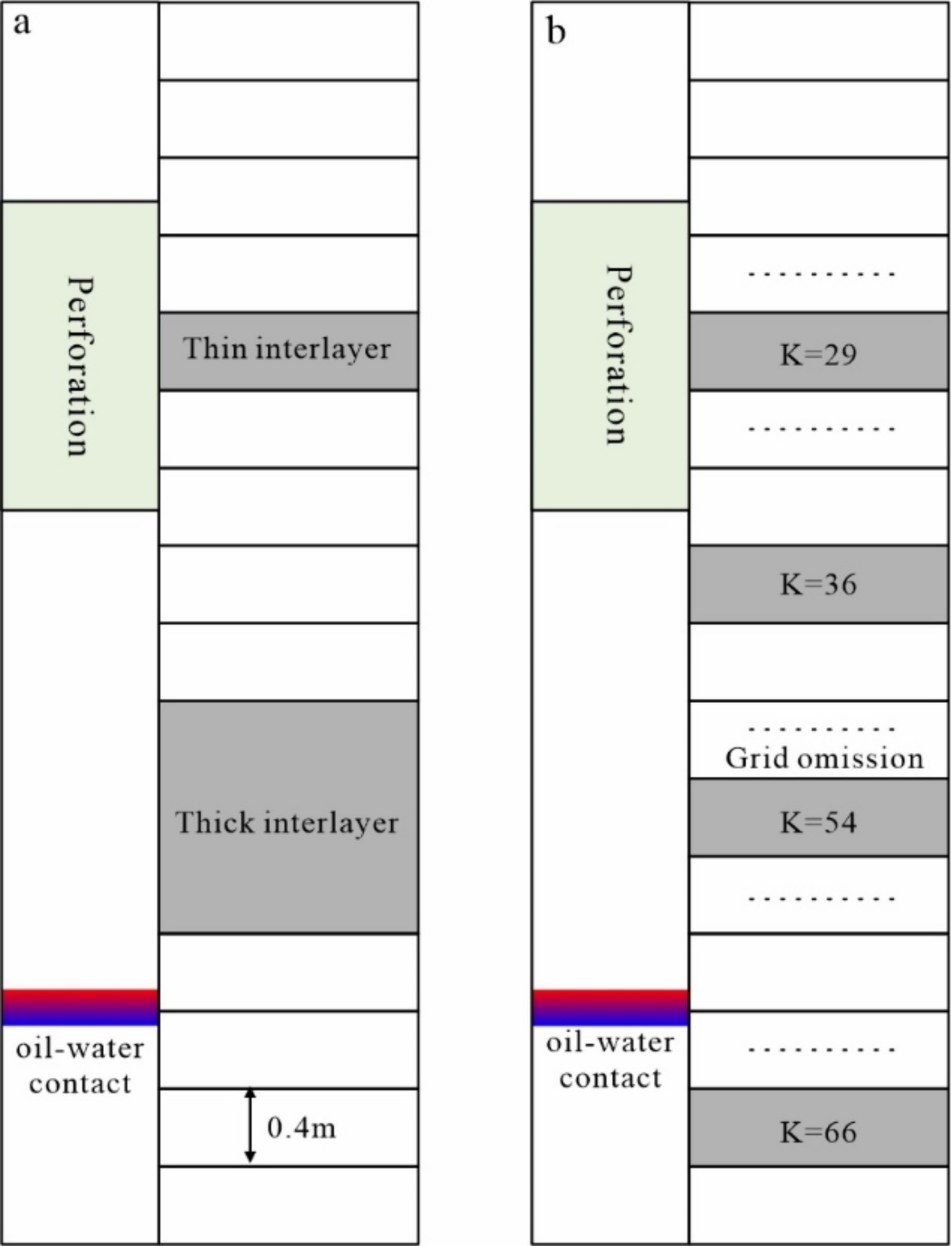
Vertical scale and position of the interlayer. (a) Thickness of the interlayer; (b) vertical position of the interlayer.

The thin interlayer thickness in this study was set at 0.4 m, with one grid spanning vertically (Fig. 6a). With three grids maintained vertically, the thick interlayer had a thickness of 1.2 m (Fig. 6a).

**Table 4:** Intralayer thickness statistics of sand formation TIII1 in Sangtamu oilfield.

Block	Interlayer thickness (m)	Block	Interlayer thickness (m)	Block	Interlayer thickness (m)	Block	Interlayer thickness (m)	Block	Interlayer thickness (m)	Block	Interlayer thickness (m)
Block-1	1	Block-1	0.37	Block-2	0.87	Block-5	0.78	Block-6	1.14	Block-6	0.82
Block-1	0.87	Block-1	0.72	Block-2	1.14	Block-5	0.89	Block-6	0.39	Block-6	0.42
Block-1	0.37	Block-2	0.25	Block-2	0.37	Block-5	0.36	Block-6	0.42	Block-6	0.58
Block-1	0.94	Block-2	0.36	Block-2	1.91	Block-5	1.24	Block-6	0.37	Block-6	2.73
Block-1	0.92	Block-2	0.99	Block-2	0.27	Block-5	0.89	Block-6	0.5	Block-6	0.62
Block-1	0.84	Block-2	0.62	Block-2	0.24	Block-5	0.75	Block-6	1.01	Block-7	1.61
Block-1	1.12	Block-2	0.26	Block-2	0.61	Block-5	1.03	Block-6	0.37	Block-7	0.89
Block-1	1.52	Block-2	1	Block-2	0.49	Block-5	1	Block-6	0.36	Block-7	0.51
Block-1	0.63	Block-2	0.37	Block-4	0.62	Block-5	1.02	Block-6	0.52	Block-7	2.29
Block-1	2.02	Block-2	1.12	Block-4	0.48	Block-5	0.47	Block-6	0.27	Block-7	1.37
Block-1	0.77	Block-2	1.12	Block-5	0.64	Block-5	0.39	Block-6	1.25	Block-7	1.02
Block-1	0.24	Block-2	1.51	Block-5	0.62	Block-5	0.5	Block-6	0.75	Block-7	0.99
Block-1	0.5	Block-2	0.56	Block-5	0.83	Block-5	0.47	Block-6	0.76	Block-7	0.63
Block-1	0.38	Block-2	0.39	Block-5	0.72	Block-5	0.47	Block-6	0.57	Block-7	1.13
Block-1	0.6	Block-2	0.63	Block-5	0.5	Block-5	0.64	Block-6	0.58	Block-7	0.63
Block-1	0.5	Block-2	0.38	Block-5	0.72	Block-5	0.64	Block-6	0.41	Block-7	0.93

#### Interlayer position

The interlayer’s vertical development position significantly influences the bottom water’s oil displacement effect and directly impacts the time required for anhydrous oil recovery in oil wells. The interlayer is located above the oil-water interface and can be roughly divided into three situations depending on its position with perforation or oil-water interface. Three distributions of the interlayer are observed: (i) inside the perforation; (ii) outside the perforation and closer to the bottom of the perforation, where the interlayer has a certain inhibitory effect on the water cone upon reaching it; (iii) outside the perforation but closer to the oil-water interface (Fig. 6a). This type of interlayer can inhibit the rising rate of the water cone, prolonging the water-free production period in the oil well and improving the recovery rate. The interlayer is located below the oil-water contact and only plays a certain role in the upward migration of formation water below it (Fig. 6b).

The vertical grid of the model consists of 80 layers in total, with layers 61–80 being water and layers 1–60 being oil (Fig. 6b). For this study’s tests, the following distributions of thin interlayers were determined: those distributed inside the perforation were set up in the 29th layer (K = 29); those distributed outside the perforation and near the bottom of the perforation were positioned in the 36th layer (K = 36); those distributed outside the perforation and near the oil-water interface were inserted in the 54th layer (K = 54); and those distributed below the oil-water interface were placed in the 66th layer (K = 66). The thick interlayer is positioned in the middle of the vertical distribution layer of the thin interlayer, with one single layer extending upwards and downwards. Thick interlayers in are located layers 28–30, 35–37, 53–55, and 65–67, respectively (Fig. 6a).

#### Interlayer shape

**Fig. 7:**
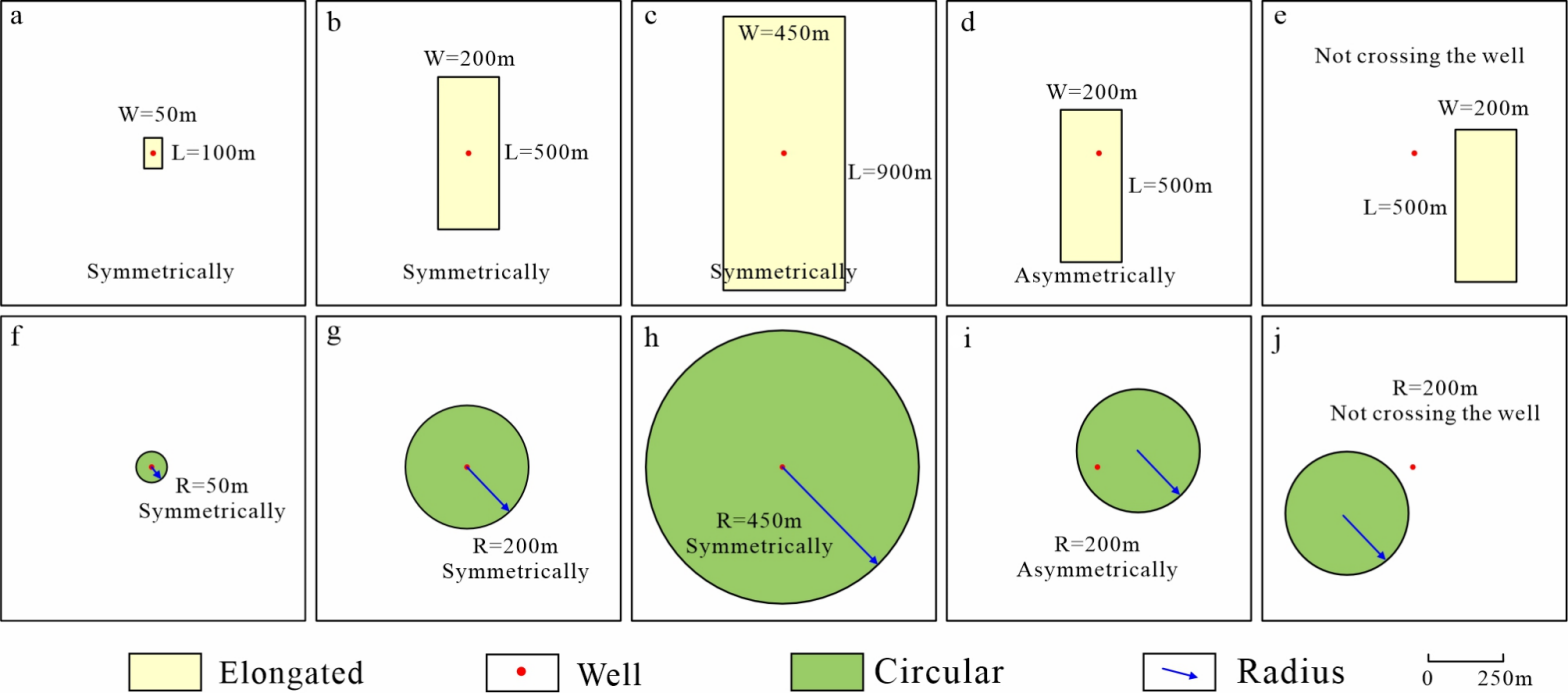
Interlayer plane scale and shape. (a) Minor-symmetric-elongated interlayer; (b) Medium-symmetric-elongated interlayer; (c) Large-symmetric-elongated interlayer; (d) Medium-asymmetric-elongated interlayer; (e) Medium-not crossing the well-elongated interlayer; (f) Small-symmetric-circular interlayer; (g) Medium-symmetric-circular interlayer; (h) Large-symmetric-circular interlayer; (i) Medium-asymmetric-circular interlayer; (j) Medium-not crossing the well-circular interlayer (where L = Length, W = Width, R = Radius).

Differences in the interlayer’s width and length on the plane result from variations in its spreading laws along the long and short-axis directions. Due to the influence of hydrodynamic conditions, it is nearly impossible to maintain the entire interlayer’s morphology during the sedimentary evolutionary stage, which leads to excessive variances in the morphology of the reservoir’s interlayer. To analyze the bottom-water coning pattern in terms of interlayer morphology and spreading scale, this study focused on circular (Fig. 7a–e) and elongated (Fig. 7f–j) interlayers in the plane that varied in size and shape.

#### Interlayer plane scale

Bottom water coning is significantly influenced by variations in the interlayer spreading area. The study defines the range of the interlayer’s width-to-thickness ratio as 50–900 and classifies it into three types: minor, medium, and major. This range is based on previous studies on the interlayer’s width-to-thickness ratio^35^.

(i) Minor interlayer.

This kind of interlayer had width-to-thickness ratios between 50 and 200, and their plan spread area determined their classification as minor interlayers. Whereas the square interlayer’s width and length are 50 m and 100 m, corresponding to 10 and 5 grids on the plane (Fig. 7a), respectively, the circular interlayer’s radius is 50 m, corresponding to 5 grids on the plane (Fig. 7f).

(ii) Medium interlayer.

The plan spread area of this type of interlayer determines its classification as a medium interlayer, with its width-to-thickness ratio ranging between 200 and 400. The width and length of the square interlayer are 200 m and 500 m, corresponding to 50 and 20 grids on the plane (Fig. 7b), respectively, while the radius of the circular interlayer is 200 m, corresponding to 20 grids on the plane (Fig. 7g).

(iii) Major interlayer.

Based on its plan spread area, this kind of interlayer—which is configured with a width-to-thickness ratio from 400 to 900—is classified as a major interlayer. The square interlayer has a width of 450 m and a length of 900 m, corresponding to 90 and 45 grids on the plane (Fig. 7c), while the circular interlayer has a radius of 450 m, corresponding to 45 grids on the plane (Fig. 7h).

When there is no interlayer, the well’s water content increases faster at the initial stage of production, the bottom water conies rapidly, and the cumulative oil production is the lowest. The bottom-water coning rate slows down, the well’s cumulative production increases, and the water content decreases with the interlayer’s growing radius, demonstrating that the interlayer delays bottom-water coning.

#### Relationship between interlayer and well location

The distribution position of the over-well interlayer, which is classified into symmetric and asymmetric varieties, can be identified via core observation and logging curve characteristics. Although geologists often apply as many means as possible to predict the location, size, and distance from the well of inter-well interlayers, there is still quite a bit of uncertainty in their estimates. However, the water cone is also significantly impacted by the distances between the well and the interlayer^12^. Three different types of interlayers were set up regarding the relationship between the interlayer and the well locations in this analysis: (i) interlayers symmetrically distributed over the wells (Fig. 7a–c, f–h), (ii) interlayers asymmetrically distributed over the wells (Fig. 7d, i), and (iii) interlayers without passing through the well, that is, inter-well interlayers (Fig. 7e, j).

### Experimental design

Orthogonal tables were chosen based on the identified factors and the number of levels. The experiment identified six primary impact factors: the location of the interlayer, type, vertical thickness, planar scale, and distance from the well (Table 5). For a multifactorial multilevel orthogonal experiment, the number of levels of the six factors taken into consideration in this experiment varied. We designed the orthogonal table L72.2.2.2.3.3.6.1 suitable for this study (Table 6).

**Table 5:** Influence factors of interlayer on bottom water coning speed.

level	Influencing factor 1 Shape	Influencing factor 2 Thickness	Influencing factor 3 Plane scale	Influencing factor 4 Relationship with well	Influencing factor 5 Type	Influencing factor 6 Vertical location
1	Circular	Thin	Minor	Symmetric	Physical	K=29
2	Elongated	Thick	Medium	Asymmetric	Muddy	K=36
3	—	—	Major	Not crossing the well	Calcareous	K=54
4	—	—	—	—	—	K=66

**Table 6:** Orthogonal experiment table.

L72.2.2.3.3.6.1																				
Number	Factor 1 2 levels	Factor 2 2 levels	Factor 3 3 levels	Factor 4 3 levels	Factor 5 3 levels	Factor 6 4 levels	Number	Factor 1 2 levels	Factor 2 2 levels	Factor 3 3 levels	Factor 4 3 levels	Factor 5 3 levels	Factor 6 4 levels	Number	Factor 1 2 levels	Factor 2 2 levels	Factor 3 3 levels	Factor 4 3 levels	Factor 5 3 levels	Factor 6 4 levels
1	1	1	1	1	1	1	25	1	2	1	3	2	2	49	2	1	1	3	2	3
2	1	1	1	1	3	4	26	1	2	2	1	3	2	50	2	1	2	1	3	3
3	1	1	2	2	1	4	27	1	2	3	2	1	2	51	2	1	3	2	1	3
4	1	1	2	2	2	1	28	1	2	1	2	3	2	52	2	1	1	2	2	2
5	1	1	3	3	2	4	29	1	2	1	3	2	1	53	2	1	2	3	3	2
6	1	1	3	3	3	1	30	1	2	2	1	3	1	54	2	1	3	1	1	2
7	1	1	1	3	1	2	31	1	2	2	3	1	2	55	2	2	1	1	3	2
8	1	1	2	1	2	2	32	1	2	3	1	2	2	56	2	2	2	2	1	2
9	1	1	3	2	3	2	33	1	2	3	2	1	1	57	2	2	3	3	2	2
10	1	1	1	1	1	4	34	1	2	1	2	3	4	58	2	2	1	2	2	4
11	1	1	2	2	2	4	35	1	2	2	3	1	4	59	2	2	2	3	3	4
12	1	1	3	3	3	4	36	1	2	3	1	2	4	60	2	2	3	1	1	4
13	1	1	1	1	3	3	37	2	1	1	2	3	1	61	2	2	1	1	1	1
14	1	1	2	2	1	3	38	2	1	2	3	1	1	62	2	2	2	2	2	1
15	1	1	3	3	2	3	39	2	1	3	1	2	1	63	2	2	3	3	3	1
16	1	1	1	2	2	1	40	2	1	1	2	3	3	64	2	2	1	3	1	3
17	1	1	2	3	3	1	41	2	1	1	3	2	4	65	2	2	2	1	2	3
18	1	1	3	1	1	1	42	2	1	2	1	3	4	66	2	2	3	2	3	3
19	1	2	1	2	2	3	43	2	1	2	3	1	3	67	2	2	1	1	1	4
20	1	2	2	3	3	3	44	2	1	3	1	2	3	68	2	2	1	1	3	1
21	1	2	3	1	1	3	45	2	1	3	2	1	4	69	2	2	2	2	1	1
22	1	2	1	3	1	3	46	2	1	1	3	1	2	70	2	2	2	2	2	4
23	1	2	2	1	2	3	47	2	1	2	1	2	2	71	2	2	3	3	2	1
24	1	2	3	2	3	3	48	2	1	3	2	3	2	72	2	2	3	3	3	4

For this examination, a total of 72 sets of comparison tests were designed (Table 6). Through the results of the experiments, the impact of various type interlayers on controlling the water breakthrough curve style features and bottom water cone inlet effect will be analyzed.

### Analysis of water breakthrough curve characteristics of different types of interlayers

**Fig. 8:**
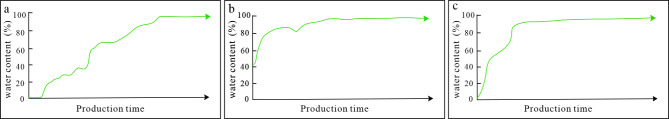
Obtaining and classifying rising patterns of water breakthrough curves based on an orthogonal experimental design. (a) Water breakthrough curve pattern for class 3 well; (b) Water breakthrough curve pattern for class 2 well; (c) Water breakthrough curve pattern for class 1 well.

The production pattern of producing wells in the TIII1 sand formation of the Sangtamu Oilfield was divided into three main groups: class 1 wells, class 2 wells, and class 3 wells based on an analysis of the characteristics of the water breakthrough curves of various interlayer types (Fig. 8). Among them, class 1 wells have the highest cumulative oil production, a longer period of anhydrous oil recovery, a slower increase in water content, and the best production effects out of all of them. Class 3 wells have the lowest cumulative oil production, rapidly increasing water content, and almost no anhydrous recovery periods. The characteristics of the water breakthrough curves for class 2 wells are midway between the previously mentioned class 1 and class 3 wells.

## Results

### Production well water breakthrough curve pattern inversion interlayer scale

Static data, including modern sedimentary and field outcrop dissections, flume simulations, and numerical simulation experiments, were primarily used to estimate the scale of the interlayer in the prior modeling procedure. The experimental test aims to combine the dynamic and static data to generate a quantitative geologic knowledge database of the interlayer parameters of the TIII1 Sand Formation in the Sangtamu Oilfield. The inversion of the interlayer scale based on the water breakthrough curve established in this manuscript is shown in Table 7.

**Table 7:** Interlayer scale based on water breakthrough curve inversion.

Thickness	Type	Vertical position	Relationship with Well	Water curve style	Estimated interlayer scale (m)	Thickness	Type	Vertical position	Relationship with Well	Water curve style	Estimated interlayer scale (m)
Thin interlayer	Physical interlayer	Within perforation	Symmetrical	Class 2	50–200	Thick interlayer	Physical interlayer	Within perforation	Symmetrical	Class 2	200-300
			Asymmetrical	Class 1	50–150				Asymmetrical	Class 2	300-450
			Interwell	—	—				Interwell	Class 1	150-350
		Outside perforation, near perforation	Symmetrical	Class 3	250–450			Outside perforation, near perforation	Symmetrical	Class 3	150-300
			Asymmetrical	Class 3	350–450				Asymmetrical	Class 3	250-450
			Interwell	Class 2	300–350				Interwell	Class 2	100-250
		Outside perforation, near oil-water contant	Symmetrical	Class 1	350–450			Outside perforation, near oil-water contant	Symmetrical	Class 1	150-350
			Asymmetrical	—	—				Asymmetrical	Class 1	200-450
			Interwell	—	—				Interwell	Class 1	250-400
		Aquifer	Symmetrical	—	—			Aquifer	Symmetrical	—	—
			Asymmetrical	—	—				Asymmetrical	—	—
			Interwell	—	—				Interwell	—	—
	Muddy interlayer	Within perforation	Symmetrical	Class 1	50–200		Muddy interlayer	Within perforation	Symmetrical	Class 2	150-250
			Asymmetrical	Class 1	150–350				Asymmetrical	Class 2	150-300
			Interwell	—	—				Interwell	Class 1	50-100
		Outside perforation, near perforation	Symmetrical	Class 3	150–350			Outside perforation, near perforation	Symmetrical	Class 3	200-450
			Asymmetrical	Class 3	200–400				Asymmetrical	Class 3	300-450
			Interwell	Class 2	250–350				Interwell	Class 1	50-150
		Outside perforation, near oil-water contant	Symmetrical	Class 1	200–350			Outside perforation, near oil-water contant	Symmetrical	Class 1	50-200
			Asymmetrical	Class 1	300–450				Asymmetrical	Class 1	50-200
			Interwell	Class 1	200–300				Interwell	Class 1	100-300
		Aquifer	Symmetrical	Class 1	150–250			Aquifer	Symmetrical	Class 1	100-200
			Asymmetrical	Class 1	250–450				Asymmetrical	Class 1	150-200
			Interwell	—	—				Interwell	—	—
	Calcareous interlayer	Within perforation	Symmetrical	Class 1	50–150		Calcareous interlayer	Within perforation	Symmetrical	Class 3	150-300
			Asymmetrical	Class 1	150–250				Asymmetrical	Class 3	200-400
			Interwell	Class 1	150–350				Interwell	Class 2	150-300
		Outside perforation, near perforation	Symmetrical	Class 3	100–300			Outside perforation, near perforation	Symmetrical	Class 3	50-250
			Asymmetrical	Class 3	250–450				Asymmetrical	Class 3	200-300
			Interwell	Class 3	300–450				Interwell	Class 3	150-450
		Outside perforation, near oil-water contant	Symmetrical	Class 1	200–400			Outside perforation, near oil-water contant	Symmetrical	Class 1	100-250
			Asymmetrical	Class 1	250–450				Asymmetrical	Class 1	150-350
			Interwell	Class 1	250–450				Interwell	Class 1	150-250
		Aquifer	Symmetrical	Class 1	150–300			Aquifer	Symmetrical	Class 1	50-250
			Asymmetrical	Class 1	350–450				Asymmetrical	Class 1	50-250
			Interwell	—	—				Interwell	—	—

— Indicates that this type of interlayer has no effect on the water cone time or a small effect that is difficult to observe.

### Static data acquisition interlayer scale

The scale characteristics of the interlayer in the study area were obtained comprehensively by combining the lithofacies characteristics and logging curves of the TIII1 sand formation in the Sangtamu Oilfield, applying the theory of comparative sedimentology, and interpreting the similar outcrops and modern sedimentary architecture. We have done much work to obtain interlayer scales based on various static data^29^. The method of obtaining these data is not the focus of this paper, so we will not go into detail here. Table 8 shows the partial interlayer scales based on field outcrop measurements.

**Table 8:** Interlayer width and thickness based on outcrop measurements.

Microfacies types	Real measurement width (m)	Estimate real width (m)	Thickness (m)
Interlayer	57.95	144.88	0.49
Interlayer	112.41	112.41	1.47
Interlayer	244.43	733.29	2.96
Interlayer	47.67	95.34	1.13
Interlayer	128.42	256.84	0.81
Interlayer	80.53	161.06	0.35
Interlayer	59.59	59.59	0.65
Interlayer	17.51	17.51	0.31
Interlayer	57.98	57.98	0.23
Interlayer	42.12	42.12	0.15
Interlayer	36.99	55.485	0.46
Interlayer	91.74	137.61	0.37
Interlayer	79.13	79.13	0.98

### Application of LN14 well

**Fig. 9:**
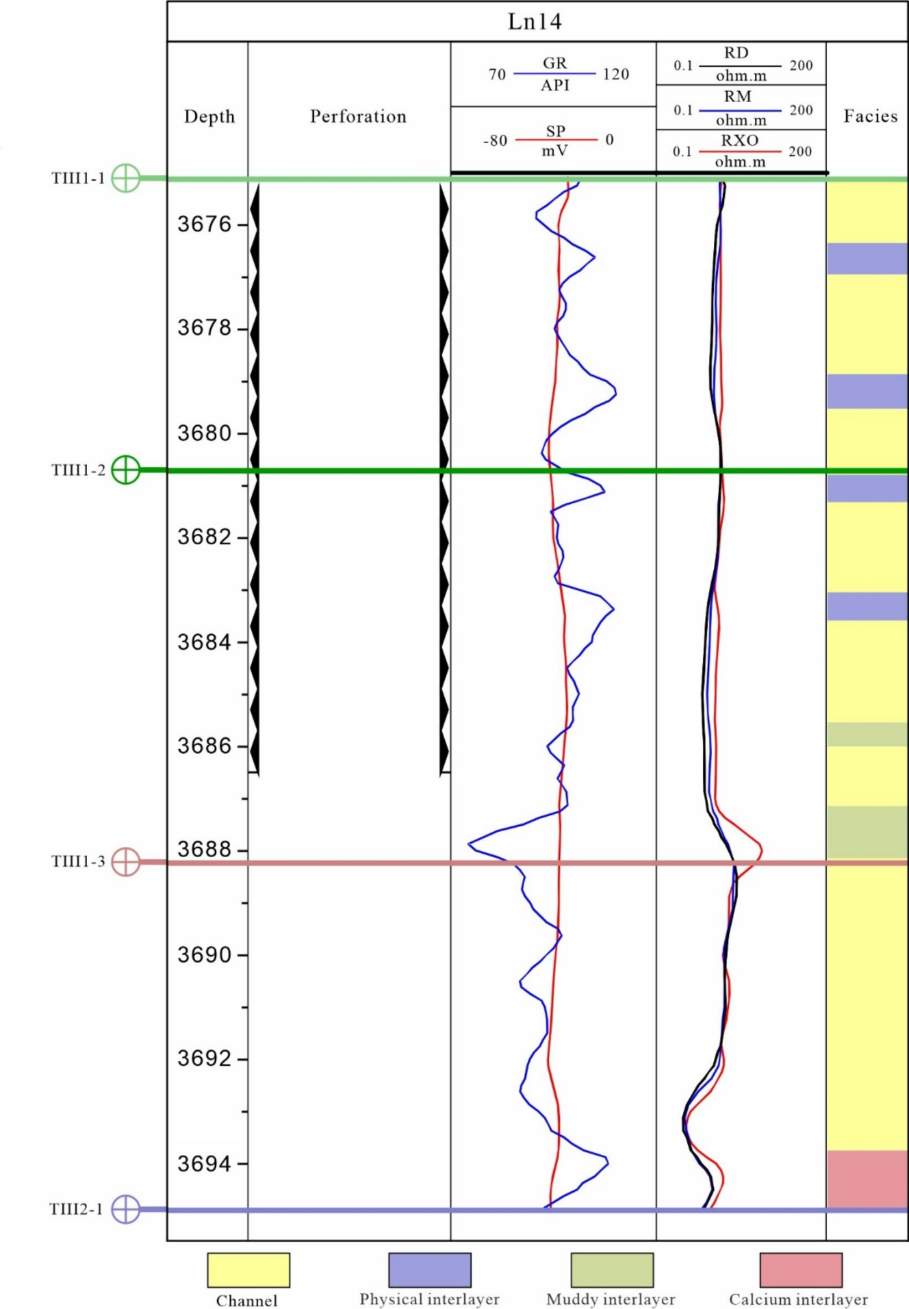
Location and thickness of interlayer in LN14 well.

For practical application, the LN14 well in the Sangtamu oil field serves as an example. The LN14 well’s perforation section is situated at 3674.49–3686.49 m. Seven interlayers (referred to as Interlayer 1-Interlayer 7 from top to bottom, respectively) were identified within the TIII1 sand formation reservoir based on the characteristics of the logging curve and core comparison (Fig. 9). These interlayers consist of two calcareous interlayers, one muddy interlayer, and four physical interlayers. The distribution location covers the interior of the perforation, the bottom of the perforation, and the vicinity of the oil-water interface.

**Fig. 10:**
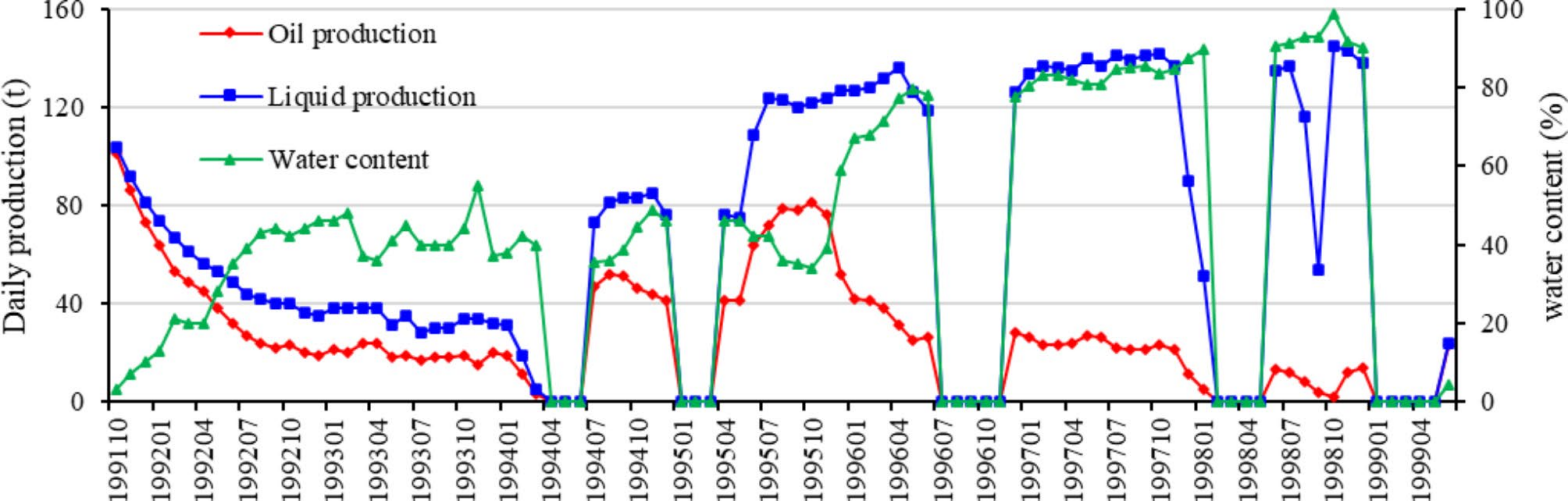
Production curve of LN14 well.

The oil and water production curves of the LN14 well indicate that the water production curve characteristics of the well should be classified as Class 2 (Fig. 10), as described in this article. Based on dynamic and static data, a quantitative geological knowledge database of the TIII1 sand formation interlayers in the Sangtamu Oilfield was established, and the scale of seven interlayers in the LN14 well was obtained (Table 9). From top to bottom, the plane scales of interlayer 1 to interlayer 7 are 100 m, 50 m, 150 m, 150 m, 50 m, 100 m, and 150 m, respectively.

**Table 9:** LN14 well interlayer scale.

Interlayer	Interlayer types	Thickness (m)	Interlayer position	Plane scale (m)
1	Physical interlayer	0.61	Inside the perforation	100
2	Physical interlayer	0.88	Inside the perforation	50
3	Physical interlayer	0.52	Inside the perforation	150
4	Physical interlayer	0.56	Inside the perforation	150
5	Muddy interlayer	0.47	Inside the perforation	50
6	Muddy interlayer	1.02	Outside the perforation, and closer to the bottom of the perforation	100
7	Calcium interlayer	1.13	Outside the perforation, but closer to the oil-water interface	150

## Discussion

**Fig. 11:**
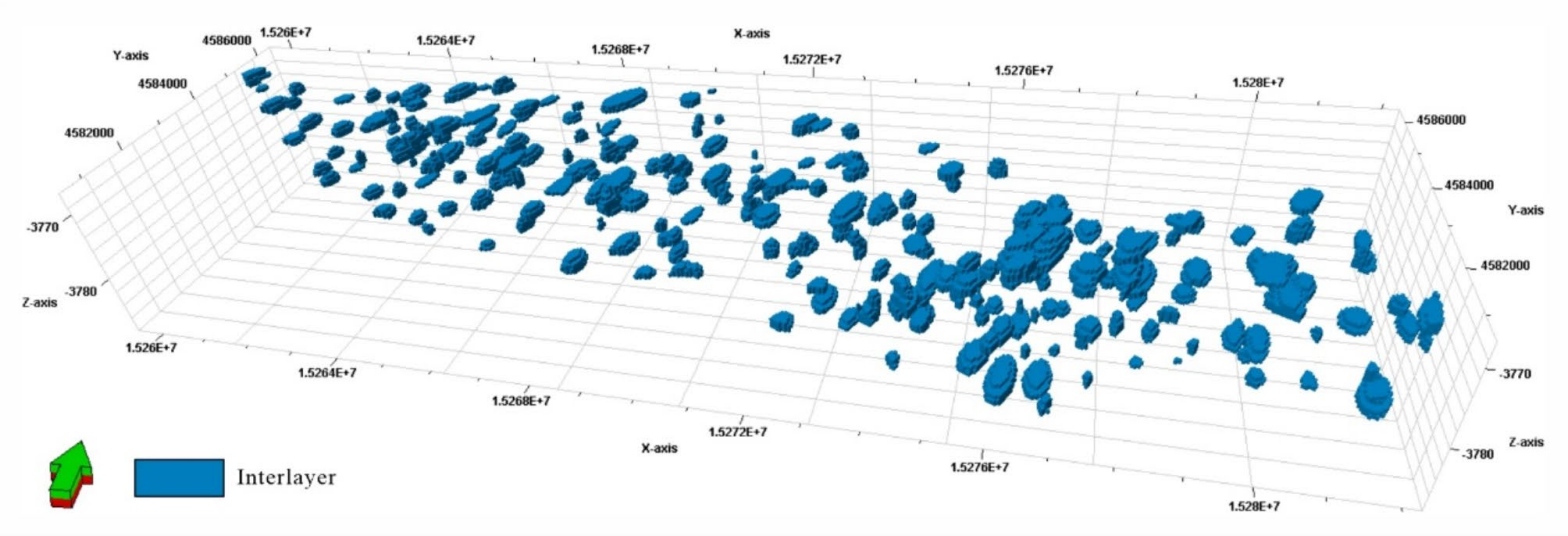
Interlayer model established based on geological knowledge database (The vertical was magnified 150 times to visualize the interlayer morphology).

Employing the interlayer scales that correspond to the characteristics of various water curves in the geologic knowledge database, a target-based method was used to model the Sangtamu oilfield interlayer (Fig. 11). The variance function analysis of the 3D geologic model results showed the following results: a width-to-thickness ratio ranging from about 300–600; a major range of 794.06 m; a minor range of 412.34 m; and a vertical range of 1.336 m. The results match the scale range of the interlayer parameters measured based on conventional static geologic data, which validates the reliability of the inversion of the interlayer scale method based on the dynamic data mentioned in this paper. This study offers an unusual viewpoint on estimating the interlayer’s scale while providing an acceptable strategy for creating the interlayer model for various depositional systems.

**Fig. 12:**
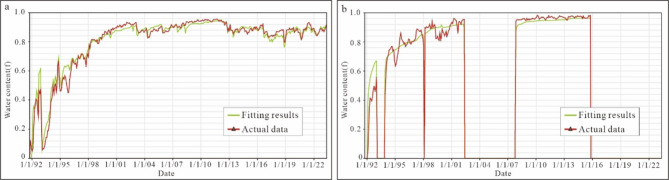
Reservoir dynamics fitting results. (a) Overall water content of TIII oil formation; (b) Water content fitting of well LN22.

**Fig. 13:**
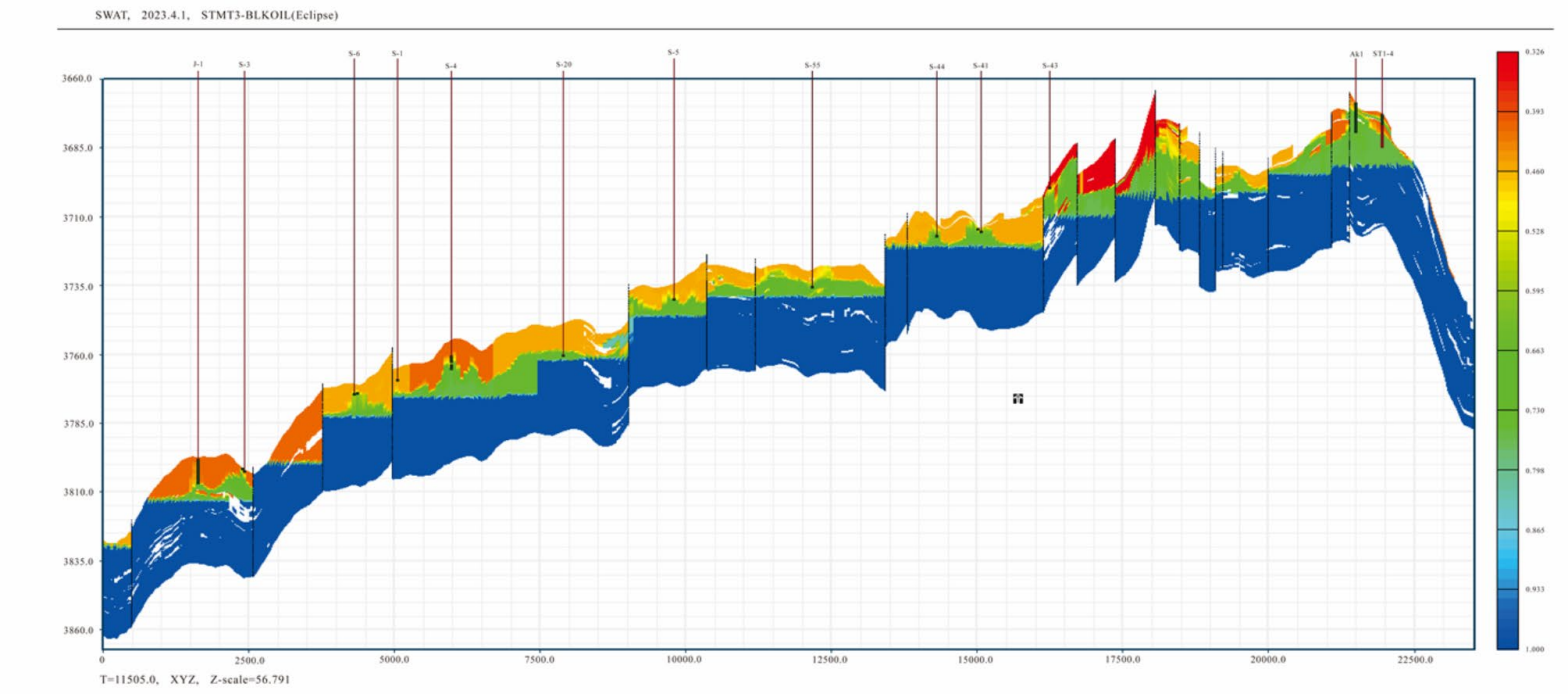
Distribution of remaining oil profile in TIII oil formation.

The numerical model for the TIII1 Sand Formation has a higher than 96% history matching coincidence rate (Fig. 12a) and a higher than 90% single-well matching coincidence rate (Fig. 12b). As a result of bottom water cone advancement and enrichment of remaining oil between the cones, the area with a high degree of utilization in the vertical direction of the TIII1 sand formation has mostly its remaining oil distributed at the top of the formation (Fig. 13).

## Conclusion

The method used in this paper is to apply production dynamics data from bottom water reservoirs to invert the interlayer’s scale, create a quantitative geologic knowledge database about the interlayer by combining it with geostatic data, and provide guidance on establishing a 3-D interlayer model. The accuracy of the method was verified by reservoir numerical simulation. Therefore, we propose a comparable method for determining the interlayer scale’s range in the rest of the reservoir.

1. Applying an orthogonal experimental design, we analyze the factors influencing the interlayer’s water cone velocity and classify the characteristics of the water breakthrough curves of various interlayer types.
2. Based on the combination of dynamic data from production wells and geostatic static data to obtain the scale range of interlayers, a quantitative geologic knowledge database of the interlayer of TIII1 sand formation in Sangtamu Oilfield was established.
3. The results of the 3D geological modeling guided by the geologic knowledge base were verified by numerical simulation, with a field-wide historical fit compliance rate of up to 96% and a single-well historical compliance rate of greater than 90%.

## Data Availability

All data generated or analysed during this study are included in this published article.

## References

[1] Hou, J. et al. Macroscopic three-dimensional physical simulation of water flooding in multi-well fracture-cavity unit. *Petrol. Explor. Dev.* **6**, 784–789 (2014).

[2] Zhao, X. & Zhu, S. Prediction of water breakthrough time for oil wells in low-permeability bottom water reservoirs with barrier. *Petrol. Explor. Dev.* **39**, 504–507 (2012).

[3] Wu, Y. et al. Study on the mechanism of improved oil recovery by nitrogen foam flooding in bottom water reservoirs. *Front. Energy Res.* **11**, 1120635 (2023).

[4] Nashawi, I., Al-Anzi, E. & Hashem, Y. A depletion strategy for an active bottom-water drive reservoir using analytical and numerical models—field case study. *J. Heat. Transf.* **31**, 101008 (2009).

[5] Wang, Y. et al. A visualized investigation on the mechanisms of anti-water coning process using nitrogen injection in horizontal wells. *J. Petrol. Sci. Eng.* **166**, 636–649 (2018).

[6] Xue, Y. & Cheng, L. The influence of interlayer of bottom water reservoirs during the development stage. *Pet. Sci. Technol.* **31**, 849–855 (2013).

[7] Zhang, X. & Sun, W. A study on the elimination of barrier to bottom water coning. *J. Northwest. Univ. (Nat Sci. Ed)*. **29**, 59–62 (1999).

[8] Zhao, J. et al. An intelligent identification method of interlayers in deep clastic rock—an example of Donghe Sandstone in Hade Oilfield, Tarim Basin. *Mar. Petrol. Geol.* **156**, 106419 (2023).

[9] Dou, M. et al. Reservoir modeling of braided river reservoirs based on geological knowledge database: a case study of P1x formation of the Daniudi Gas Field, Ordos Basin, China. *Lithos Spec.* **13**, 6913641 (2022).

[10] Xu, F., Huang, W., Meng, Z., Sun, T. & Zhang, W. Barrier/interbed quantitative characterization and modeling in M block in orinoco heavy-oil belt. In *Proceedings of the International Field Exploration and Development Conference, 2019* 2318–2327 (2020).

[11] Li, Y., Wu, S., Hou, J. & Liu, J. Progress and prospects of reservoir development geology. *Petrol. Explor. Dev.* **44**, 603–614 (2017).

[12] Hu, Y. et al. The effect of interlayer on water cut rise in a bottom water reservoir. *Geofluids* **2021**, 1–9 (2021).

[13] Yue, P., Jia, B., Sheng, J., Lei, T. & Tang, C. A coupling model of water breakthrough time for a multilateral horizontal well in a bottom water-drive reservoir. *J. Petrol. Sci. Eng.* **177**, 317–330 (2019).

[14] Wang, Y., Chen, X., Zou, L., Yang, B. & Wang, Y. Analysis on oil wells bottom water coning influence factors of bottom water reservoir with interlaye. *Petrol. Reserv. Eval Dev.* **3**, 24–27 (2013).

[15] Guo, X. et al. Gas-well water breakthrough time prediction model for high-sulfur gas reservoirs considering sulfur deposition. *J. Petrol. Sci. Eng.* **157**, 999–1006 (2017).

[16] Zhang, P., Zhang, Y., Zhang, W. & Tian, S. Numerical simulation of gas production from natural gas hydrate deposits with multi-branch wells: influence of reservoir properties. *Energy* **238**, 121738 (2022).

[17] Wang, Y., Voskov, D., Khait, M. & Bruhn, D. An efficient numerical simulator for geothermal simulation: a benchmark study. *Appl. Energy*. **264**, 114693 (2020).

[18] Zhang, K., Jin, Z. & Li, S. Coupled miscible carbon utilization-storage processes in fractured shales. *Chem. Eng. J.* **441**, 135987 (2022).

[19] Sun, X. et al. Numerical simulation of gas recovery from a low-permeability hydrate reservoir by depressurization. *Appl. Energy*. **250**, 7–18 (2019).

[20] Yuan, Q. Water breakthrough time prediction of oil wells in the bottom water reservoir. *Petrol. Geol. Eng.* **32**, 73–75 (2018).

[21] Trivedi, J. & Babadagli, T. Experimental and numerical modeling of the mass transfer between rock matrix and fracture. *Chem. Eng. J.* **146**, 194–204 (2009).

[22] He, X. et al. Architecture of shallow-water delta reservoir of Huagang formation in C Oilfield, Xihu Sag. *Xinjiang Petrol. Geol.* **44**, 517–527 (2023).

[23] Niu, B. et al. Hierarchical modeling method based on multilevel architecture surface restriction and its application in point-bar internal architecture of a complex meandering river. *J. Petrol. Sci. Eng.* **205**, 108808 (2021).

[24] Wang, Y., Tang, Y., Gao, J. & Jiang, R. Geological modeling of interlayer in tight reservoir: a case study of Chang 6 member in Chaishangyuan area of Qilicun Oifield. *J. Xi’an Shiyou Univ. (Nat Sci. Ed)*. **37**, 27–33 (2022).

[25] Cheng, H., Hou, G. & Gong, F. The interlayer causes and the recognition method of fan delta front sand-conglomerate reservoir. *Xinjiang Geol.* **31**, 269–273 (2013).

[26] Niu, B. et al. Trend judgment of abandoned channels and fine architecture characterization in meandering river reservoirs: a case study of Neogene Minhuazhen formation NmIII2 layer in Shijiutuo bulge, chengning uplift, Bohai Bay Basin, East China. *Petrol. Explor. Dev.* **46**, 943–953 (2019).

[27] Liu, Z., Lyu, W., Liao, X., Zhu, J. & Gao, H. Geostatistical inversion-based fine characterization of interlayers in braided river reservoirs for horizontal well pattern: taking Guantao formation of LD-A oilfield in Liaozhong Sag, Bohai Sea as an example. *China Offshore Oil Gas* **34**, 71–81 (2022).

[28] Fu, C. et al. Types and sedimentary genesis of barriers and interlayers in the composite turbidite sand bodies of deep-water canyon: a case study of the Central Canyon in the Qiongdongnan Basin. *Nat. Gas Ind.* **43**, 23–33 (2023).

[29] Leeder, M. Fluviatile fining-upwards cycles and the magnitude of palaeochannels. *Geol. Mag*. **110**, 265–276 (1973).

[30] Lorenz, J., Clark, J., Heinze, D. & Searls, C. Determination of widths of meander-belt sandstone reservoirs from vertical downhole data, Mesaverde Group, Piceance Creek Basin, Colorado. *AAPG Bull.* **69**, 710–721 (1985).

[31] Dou, M. et al. Combined synchronous simulation of discrete and continuous variables under high net-to-gross ratio reservoir. *Geoenergy Sci. Eng.* **229**, 212071 (2023).

[32] Lyu, D. et al. Seepage characterization based on sandstone particle motion pattern: a case study of the TIII reservoir in the Sangtamu Oilfield. *Geofluids* **2022**, 2084431 (2022).

[33] Pang, Z., Wang, L., Yin, F. & Lyu, X. Steam chamber expanding processes and bottom water invading characteristics during steam flooding in heavy oil reservoirs. *Energy* **234**, 121214 (2021).

[34] Feng, C. et al. Reservoir architecture and remaining oil distribution of deltaic front underwater distributary channel. *Petrol. Explor. Dev.* **41**, 358–364 (2014).

[35] Tong, Q. et al. Architecture characterization of single distributary channel sand bodies restricted by architecture interface of Yan 8 member in Y116 well area, Yanwu Oilfield. *Lithol. Reserv.* **32**, 144–158 (2020).

